# Copper-binding protein modelling by single-cell transcriptome and Bulk transcriptome to predict overall survival in lung adenocarcinoma patients

**DOI:** 10.7150/jca.94588

**Published:** 2024-03-17

**Authors:** Min Shengping, Wang Luyao, Xie Yiluo, Chen Huili, Wang Ruijie, Song Ge, Wang Xiaojing, Lian Chaoqun

**Affiliations:** 1 Anhui Province Key Laboratory of Clinical and Preclinical Research in Respiratory Disease, The Department of Pulmonary Critical Care Medicine, First Affiliated Hospital of Bengbu Medical University, Bengbu, 233030, China.; 2 Department of Genetics, School of Life Sciences, Bengbu Medical University, Bengbu, 233030, China.; 3 Department of Clinical Medicine, Bengbu Medical University, Bengbu, 233030, China.; 4 Research Center of Clinical Laboratory Science, Bengbu Medical University, Bengbu, 233030, China.; 5 Molecular Diagnosis Center, Joint Research Center for Regional Diseases of IHM, The First Affiliated Hospital of Bengbu Medical University, Bengbu, 233030, China.; 6 Department of Stomatology, Bengbu Medical University, Bengbu, 233030, China.

**Keywords:** Lung adenocarcinoma, Copper-Binding, Single-cell RNA-seq, Prognosis, Immunotherapy efficacy

## Abstract

**Background:** Copper and copper-binding proteins are key components of tumour progression as they play an important role in tumour invasion and migration, and abnormal accumulation of copper (Cu) may be intimately linked to with lung adenocarcinoma (LUAD).

**Methods:** Data on lung adenocarcinoma were sourced from the Cancer Genome Atlas (TCGA) database and the National Centre for Biotechnology Information (GEO). 10x scRNA sequencing, which is from Bischoff P et al, was used for down-sequencing clustering and subgroup identification using TSNE. The genes for Copper-binding proteins (CBP) were acquired from the MSigDB database. LASSO-Cox analysis was subsequently used to construct a model for copper-binding proteins (CBPRS), which was then compared to lung adenocarcinoma models developed by others. External validation was carried out in the GSE31210 and GSE50081 cohorts. The effectiveness of immunotherapy was evaluated using the TIDE algorithm and the IMvigor210, GSE78220, and TCIA cohorts. Furthermore, differences in mutational profiles and the immune microenvironment between different risk groups were investigated. The CBPRS's key regulatory genes were screened using ROC diagnostic and KM survival curves. The differential expression of these genes was then verified by RT-qPCR.

**Results:** The six CBP genes were identified as highly predictive of LUAD prognosis and significantly correlated with it. Multivariate analysis showed that patients in the low-risk group had a higher overall survival rate than those in the high-risk group, indicating that the model was an independent predictor of LUAD. The CBPRS demonstrated superior predictive ability compared to 11 previously published models. We constructed a column-line graph that includes CBPRS and clinical characteristics, which exhibits high predictive performance. Additionally, we observed significant differences in biological functions, mutational landscapes, and immune cell infiltration in the tumour microenvironment between the high-risk and low-risk groups. It is noteworthy that immunotherapy was also significant in both the high- and low-risk groups. These results suggest that the model has good predictive efficacy.

**Conclusions:** The CBP model demonstrated good predictive performance, revealing characteristics of the tumour microenvironment. This provides a new method for assessing the efficacy of pre-immunisation and offers a potential strategy for future treatment of lung adenocarcinoma.

## Introduction

Globally, lung cancer has been the leading cause of cancer deaths 1. Of these, lung adenocarcinoma (LUAD) is the most common histological subtype 2, 3. Lung adenocarcinoma (LUAD) is the most common pathological subtype of lung cancer, accounting for approximately 40% of all lung cancer cases 4. LUAD is the most common pathological subtype of lung cancer, accounting for approximately 40% of all lung cancer cases. Despite significant advances in combination treatment strategies for LUAD, the average 5-year survival rate for LUAD is approximately 15% 5. This calls for the discovery of new therapeutic targets and effective combination therapy strategies for LUAD.

Copper is an indispensable metal ion for the human body and plays a vital role in various physiological activities by acting as a cofactor for key enzymes involved in biological functions 6. However, tumours are also particularly dependent on the metal, as angiogenesis can be promoted 7. Elevated serum copper levels in cancer patients, as well as elevated copper levels in tumour tissues, support the possibility that cancer cells have an increased need for copper 8. A growing body of evidence reveals an important role for copper in the malignant phenotype of cancer. Copper has been reported to regulate the migratory capacity of cancer cells by activating metabolic and proliferative enzymes 8. In addition, copper has been reported to regulate the angiogenic process by increasing the expression of angiogenic factors, an important hallmark of cancer 9. In addition, copper may serve as a new target for cancer therapy 8, 10. Clinical studies have revealed the anti-cancer efficacy of several copper chelators 11. For example, tetrathiomolybdate, a copper chelator used in the treatment of Wilson's disease, a hereditary copper overload disorder, significantly inhibits angiogenesis and metastasis by targeting NF-κB signalling in breast cancer 12.

Copper-binding proteins play significant roles in tumours. However, their role in lung adenocarcinoma has not been studied yet. These proteins are crucial regulators of copper homeostasis and downstream effectors of copper. It is reasonable to speculate that copper-binding proteins may play important roles in copper-mediated tumour progression. We conducted a systematic analysis of the role of copper-binding proteins in lung adenocarcinomas. We explored their expression patterns, prognostic value, and immune microenvironmental regulation in lung adenocarcinomas. Our bioinformatic analyses generated a novel copper-binding protein-based column-line graphical model for predicting the prognosis of LUAD patients. This contributes to our understanding of the therapeutic applications of copper-binding proteins in the treatment of LUAD.

## Materials and Methods

### Copper binding protein gene set

The copper-binding protein gene set was obtained from five gene sets in MSigDB (https://www.gsea-msigdb.org/gsea/msigdb) 13. In addition, based on previous studies 14, several other copper-binding proteins were added. Ultimately, after removing overlapping genes, a total of genes were identified and are listed in **Supplement Table S1**.

### Data collection and processing

Clinical information regarding LUAD patients, along with extensive RNA sequencing results, as well as data on copy number variants (CNVs) and single nucleotide variants (SNVs), were obtained from the TCGA website 15. The GEO database (https://www.ncbi.nlm.nih.gov/) was also consulted. Raw count data were first normalised using the transcripts per million (TPM) method and then log2 transformed. Our study utilised three independent cohorts. The TCGA-LUAD cohort was used as the training dataset, while the GSE31210 (n=226) and GSE50081 (n=128) cohorts were used as the validation dataset. The TCGA-LUAD cohort was used as the training dataset, while the GSE31210 (n=226) and GSE50081 (n=128) cohorts were used as the validation dataset. The TCGA-LUAD cohort was used as the training dataset, while the GSE31210 (n=226) and GSE50081 (n=128) cohorts were used as the validation dataset. Relevant prognostic features were constructed using 500 LUAD cases from the TCGA database. The sample inclusion criteria for TCGA were 01A (Primary Tumor) type samples containing complete survival information (see Supplement table S2). CNV data were processed using the maftools R package 16. The GEO database (https://www.ncbi.nlm.nih.gov/geo/) was used to obtain external data for GSE31210 and GSE50081.

### Processing of single-cell data

The scRNA-seq dataset for lung adenocarcinoma was obtained from the article 'Single-cell RNA sequencing reverses distinct tumor microenvironmental patterns in lung adenocarcinoma' by Philip Bischoff et al. 17. Initially, we utilized the 'Seurat' R package to transform the 10× scRNA-seq data into Seurat objects. We then excluded cells of substandard quality and performed quality control (QC) by calculating the percentage of mitochondrial or ribosomal genes 18. Highly variable genes were identified for subsequent analysis. Batch effects were removed using the 'Harmony' tool. Cell clusters were constructed using the 'FindClusters' and 'FindNeighbors' functions and visualised using the 't-SNE' method. Cellular annotation was performed based on marker genes of different cell types.

### Construction and validation of a risk prediction model for copper-binding proteins

The training set data underwent LASSO regression analysis using the R package 'glmnet' to achieve optimal results. We identified 6 CBP-related genes through multiple regression Cox analysis and calculated the risk score for each patient using the following formula: riskscore = 0.233*FKBP4 + 0.12*GPC1 + 0.245*LOXL2 + 0.152*MUC2+ - 0.476*SNAI3 + 0.324*SOD1. Using the median value of the risk scores, we categorized the patients in the training group into high-risk and low-risk groups. We performed Kaplan-Meier survival analyses and constructed subject work characteristic curves (ROC). To validate the predictive power of the model, we evaluated its prognosis, sensitivity, and specificity in the test group. We then validated it in the GSE31210 and GSE50081 cohorts using the risk score formula.

### Independent prognostic analysis and column chart construction

Univariate and multivariate Cox regression analyses were conducted to investigate whether CBP characteristics could act as independent predictors of LUAD patients. Nomogram were generated using the 'rms' R package to forecast 1-, 3-, and 5-year OS in clinical patients based on age, grade, gender, stage, T-stage, and risk score. The calibration study results provide additional evidence of the accuracy of the column chart predictions.

### Functional enrichment analysis

In order to elucidate the potential biological pathways associated with CBPRS, the HALLMARK and KEGG pathways were analysed in this study using the "ClusterProfiler" 19 R package in MSigDB. The "c2.cp.kegg.v7.4.symbols.gmt" and "h.all.v2023.2.Hs.symbols" were analysed by GSVA and GSEA algorithms using MSigDB 20 and GSEA algorithms to obtain the differences in enrichment pathways between different risk groups.

### Analysis of genomic variation among CBPRS risk subgroups

Mutations in built-in tumours (MATH) is a method to quantify intra-tumour heterogeneity (ITH) based on the distribution of mutant alleles. The prognostic significance of MATH has been investigated in a variety of tumours, including head and neck, colorectal and breast cancers 21-24. The MATH score was calculated for each LUAD patient using the previously described method. Survival analyses were then performed based on their MATH scores. To investigate somatic mutations associated with CBPRS, the R package 'maftools' was used to generate waterfall plots displaying mutations in LUAD patients in both high- and low-risk groups. Furthermore, we computed the tumour mutational load (TMB) score for each patient with lung adenocarcinoma (LUAD) and investigated the correlation between high and low risk groups, TMB, and survival rates.

### Correlation analysis of CBP models with the immune microenvironment

In order to estimate the immunity score, stroma score and 22 different types of immune infiltrating cells, the R packages "ESTIMATE" and "CIBERSORT" were used 25. and "CIBERSORT" 26 R packages "ESTIMATE" and "CIBERSORT" were used. Single sample immune cell infiltration scores were also quantified using single sample gene set enrichment analysis (ssGSEA) based on the R package GSVA. Finally we compared the mRNA expression levels of immune checkpoint inhibitory molecules. TIDE was used to predict tumour immunotherapy effect 27, TIDE score data were obtained from the TIDE website (http://tide.dfci.harvard.edu/).

### Immunotherapy prediction and chemotherapy sensitivity analysis

We used a GEO immunotherapy cohort (GSE78220^28^) and the IMvigor210 cohort to study the correlation between CBP characteristics and immunotherapy. We used the "IMvigor210CoreBiologies" R package from the IMvigor210 cohort to process the data 29. The 'IMvigor210CoreBiologies' R package from the IMvigor210 cohort was utilised to process data. Furthermore, to determine immunogenicity based on immunomodulators, immunosuppressive cells, MHC molecules, and effector cells, the Immunophenoscore (IPS) algorithm was employed. The IPS score is calculated based on the unbiased gene expression of a representative cell type using a machine-learning methodology. Higher IPS scores indicate a better response to immunotherapy. The IPS scores of TCGA-LUAD patient samples were obtained from The Cancer Immunome Atlas (TCIA) database available at https://tcia.at/home.

### Cell line culture and RT-qPCR

The cells were cultured at 37 °C in an incubator with a 5% CO2 atmosphere. The normal human lung cell line 2B and the lung adenocarcinoma cells H1299 and A549 were obtained from the Chinese Academy of Sciences (Shanghai, China). Thermo Fisher Scientific (Invitrogen, USA) and Corning Inc. provided the cell culture media, plates, and dishes. The 2B, H1299, and A549 cells were detached and inoculated into 60 mm dishes overnight at an initial density of 1×10^6^ cells/well. SYBR Green qPCR mix (Vazyme, China) was subsequently used to synthesize cDNA for real-time PCR. The results were analysed using the comparative Ct method and the Ct values of each gene were normalized by the Ct reads of the corresponding GAPDH. All data are expressed as the mean ± standard deviation (SD) of three independent experiments. The primer sequences are shown in Supplementary Table 3.

### Statistical analysis

Statistical analyses were conducted using R software (version 4.2.2). The Wilcoxon test was used to compare differences between groups, while the log-rank test was used to compare Kaplan-Meier survival curves. Univariate and multivariate Cox analyses were performed to establish independent prognostic factors. All P values were two-sided, and a significance level of less than 0.05 was used.

## Results

### Genetic variation and expression of prognosis-related CBPRGs in LUAD

**Figure 1:**
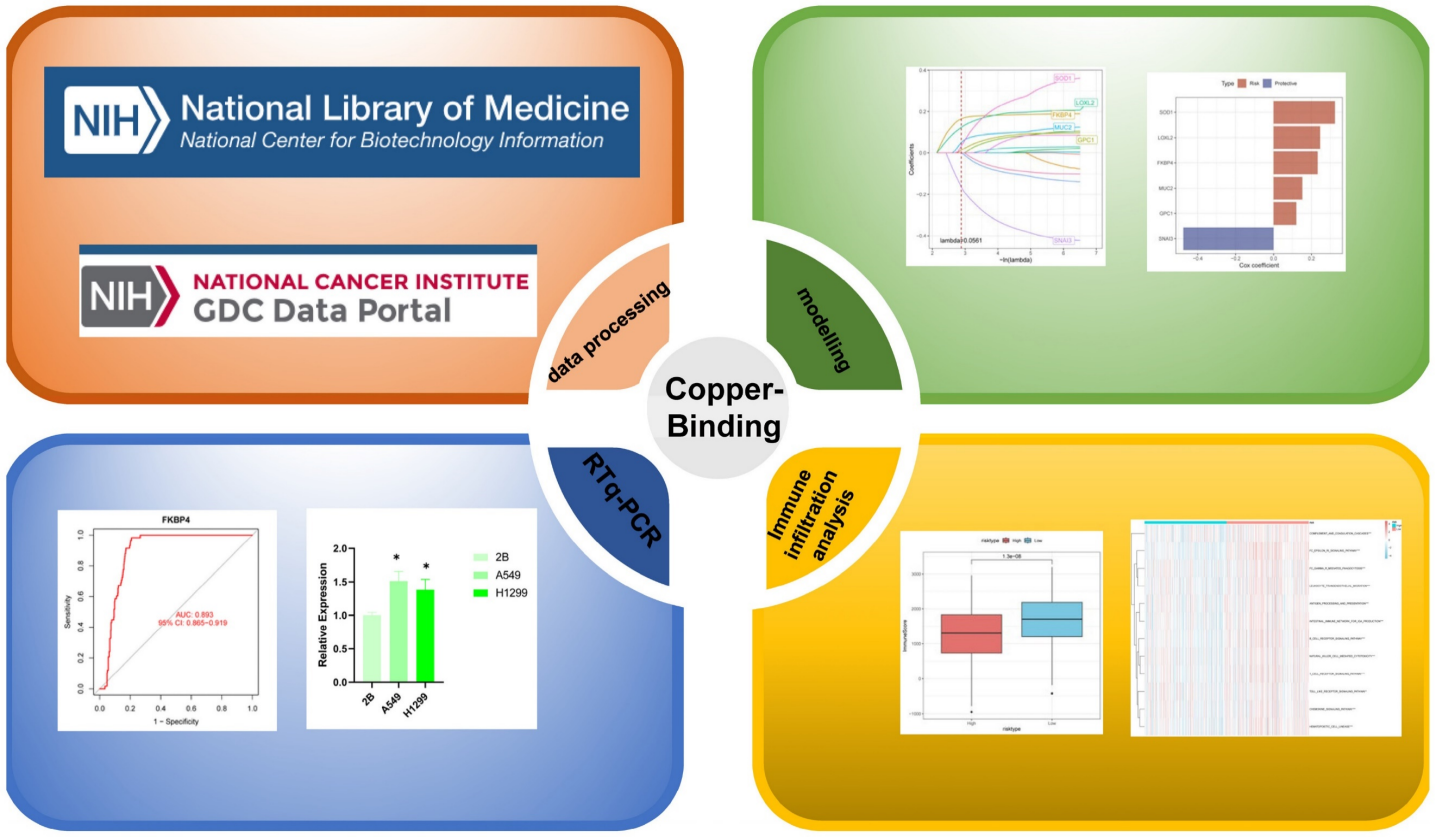
Workflow diagram of this study.

**Figure 2:**
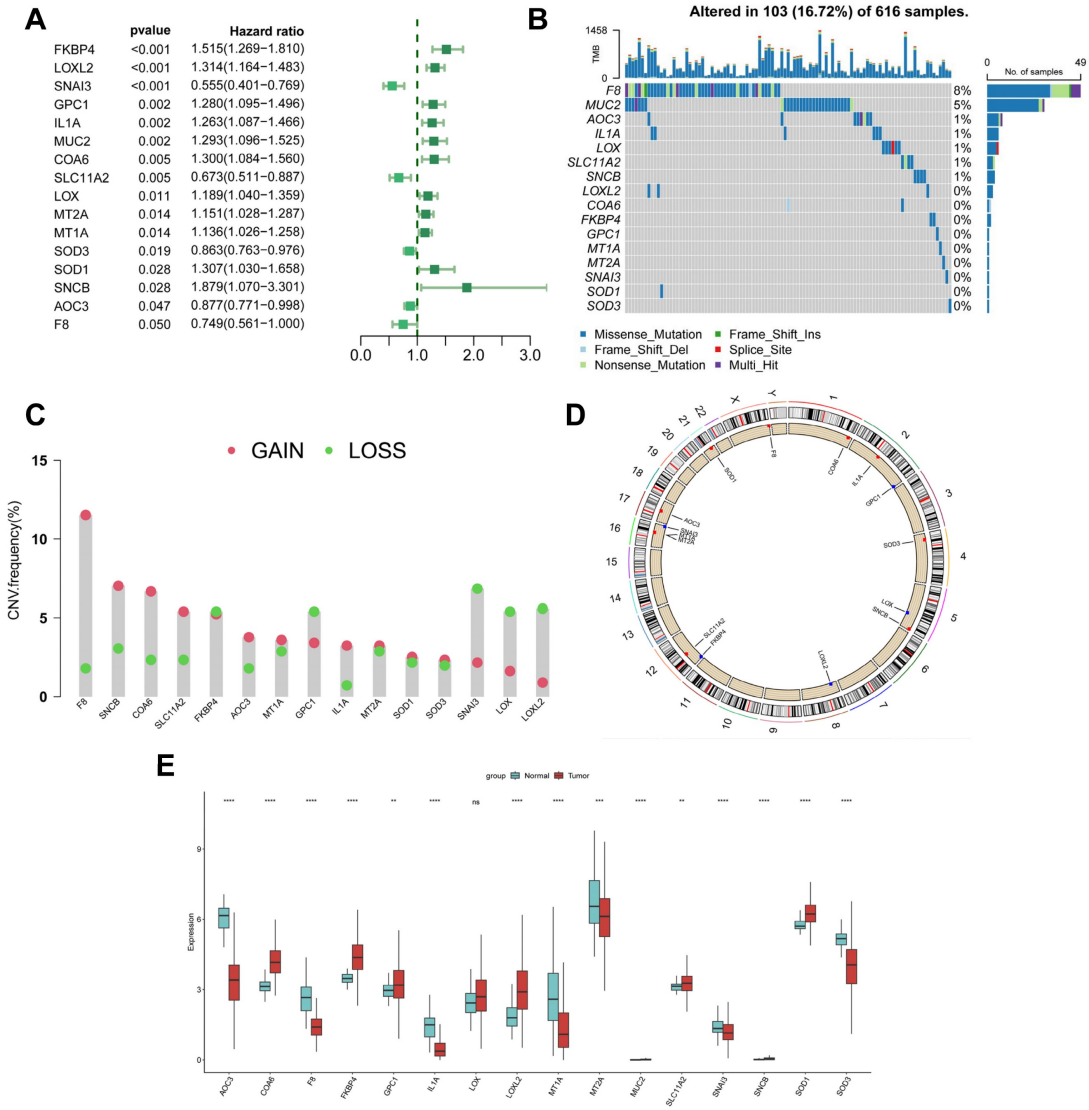
** Genetic variation and expression of CBPRGs in LUAD. (A)** Forest plot of one-way regression analysis of LUAD patients in the TCGA-LUAD dataset.** (B)** Distribution and mutation frequency of 14 CBPRGs in the TCGA-LUAD cohort. **(C)** CNV alteration frequencies of CBPRGs in LUAD, with the height of the bar representing the mutation frequency.** (D)** Location of CNV alterations in CBPRGs on chromosomes.** (E)** Expression of 14 CBPRGs genes in LUAD tumours and normal tissues ns represents not significant, *p < 0.05, **p < 0.01, ***p < 0.001, ****p < 0.0001. CBPRGs, copper bonding-related genes; LUAD, lung adenocarcinomas; TCGA, Cancer Genome mapping; CNV, copy number variation.

Figure 1 shows the workflow of our study. We collected 85 copper-binding protein genes from literature and databases. Subsequently, we screened 14 genes using univariate cox analysis, as shown in Figure 2A. We then performed copper-binding protein-related modelling (CBPRS) on these 14 genes and compared them with published article models. Our results indicate that CBPRS has good prognostic efficacy. The study examined the survival rates of CBPRS patients with various clinical factors. The results showed that patients with high CBPRS had a significantly lower prognosis than those with low CBPRS. Additionally, pathway enrichment analysis revealed that the high CBPRS group had more oncogenic pathways enriched, while the low-risk group had more immune pathways enriched. Based on the immune infiltration profile, the results indicate that the immune scores were higher in the low CBPRS group, with more immune cell infiltration. Additionally, we explored the mutational landscape in the different CBPRS groups and found a higher mutation rate in the high CBPRS group. The low CBPRS group showed better immunotherapy efficacy, as analysed by immunotherapy. Finally, two genes were screened using the ROC diagnostic curve and validated by RT-qPCR.

This study analysed 85 copper-binding protein-related genes (CBPRGs). Univariate cox regression analysis identified 14 prognostic genes associated with OS in lung adenocarcinoma patients, which are presented in Figure 2A. Mutations in CBP genes were found in 16.72% (103/616) of LUAD patients. F8 had the highest mutation rate, followed by *MUC2* and *AOC3* (Figure 2B). Somatic copy number variation (CNV) in CBPRGs was examined, revealing 14 prevalent copy number alterations in CBP. Among these, *F8*, *SNCB*, and *COA6* showed extensive CNV amplification, while CNV depletion was present in some CBP genes (Fig. 2C). The location of CNV alterations in copper-binding protein-related genes on the chromosome is demonstrated in Figure 2D. The differential expression of copper-binding protein-related genes was also explored. Through a comparison of expression levels between LUAD tumours and normal tissues, we investigated the expression of several CBPRGs in the tumours. Notably, *AOC3*, *F8*, and *IL1A* were found to be highly expressed in the normal group, while *COA6*,* FKBP4*, and *LOXL2* were highly expressed in the tumours (Figure 2E). It is important to note that the language used is clear, objective, and value-neutral, with a formal register and precise word choice. The sentence structure is simple and the logical flow of information is maintained. The technical term abbreviations are explained when first used, and the grammar, spelling, and punctuation are correct. No changes in content have been made to the original text.

### Construction and validation of CBP-related prognostic features

**Figure 3:**
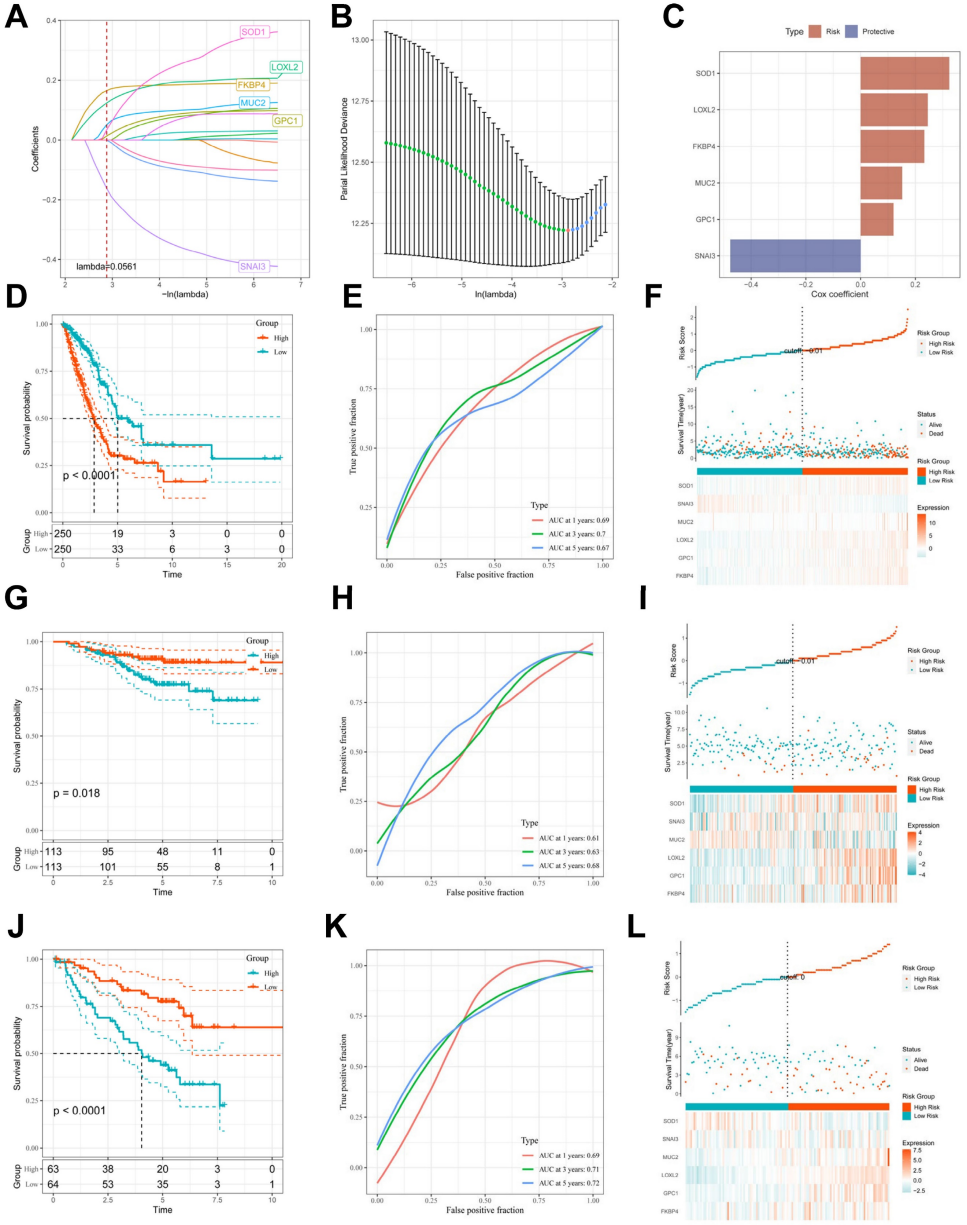
** Construction and validation of CBP-related prognostic features. (A)** Trajectories of each independent variable with lambda.** (B)** Plot of coefficient distributions generated by the logarithmic (lambda) series used for parameter selection (lambda).** (C)** Multivariate Cox coefficients for each gene in the risk profile.** (D-F)** Distributions of km curves in the TCGA-LUAD cohort, ROC curves for the CBPRS predicted risk of death at 1, 3, and 5 years, and risk scores and survival status.** (G-I)** km curves in the GSE31210 cohort, ROC curves for CBPRS-predicted risk of death at 1, 3, and 5 years, and distributions of risk scores and survival status.** (J-L)** km curves, ROC curves for CBPRS-predicted risk of death at 1, 3, and 5 years, and distribution of risk scores and survival status in the GSE50081 cohort.

To avoid overfitting and exclude co-expressed CBPRGs, we used lasso regression analysis to construct predictive prognostic models consisting of six CBPRGs: *FKBP4*, *GPC1*, *LOXL2*, *MUC2*, *SNAI3*, and *SOD1* (Figure 3A, B). We developed a linear prediction model based on the weighted regression coefficients of these six prognostically relevant CBPRGs. The riskscore was calculated as follows: riskscore = (0.233*FKBP4+0.12*GPC1+0.245*LOXL2+0.152*MUC2+-0.476*SNAI3+0.324*SOD1) (Figure 3C). To demonstrate the stability and reliable generalisation of our model, we used the TCGA-LUAD cohort as the internal training set, and the GSE31210 and GSE50081 cohorts as the external validation cohorts. Using the same risk formula, we calculated risk scores for each sample in the TCGA training cohort and the GEO validation cohort. We found that when the risk of LUAD patients was elevated in both cohorts, patients exhibited a survival disadvantage with reduced overall survival and increased mortality (see Figure 3F, I, L). Patients were classified into two subgroups, high-risk and low-risk, based on their median risk score. The prognostic differences between the two groups were explored using Kaplan-Meier curves. The curves showed significant differences in prognosis between high-risk and low-risk patients in both cohorts. Patients in the low-risk group experienced a more pronounced survival advantage (see Figure 3D, G, J). The ROC curves were used to predict the patient's 1-year, 3-year, and 5-year survival times. The AUCs for the TCGA-LUAD cohort were 0.69, 0.70, and 0.67, for the GSE31210 cohort were 0.61, 0.63, and 0.68, and for the GSE50081 cohort were 0.69, 0.71, and 0.73 (Figure 3E, H, K), respectively. These results suggest that the model has a good predictive effect. The clinical trilinear table can be found in Table 1.

### Creation of nomogram based on CBP models combined with clinical features

**Figure 4:**
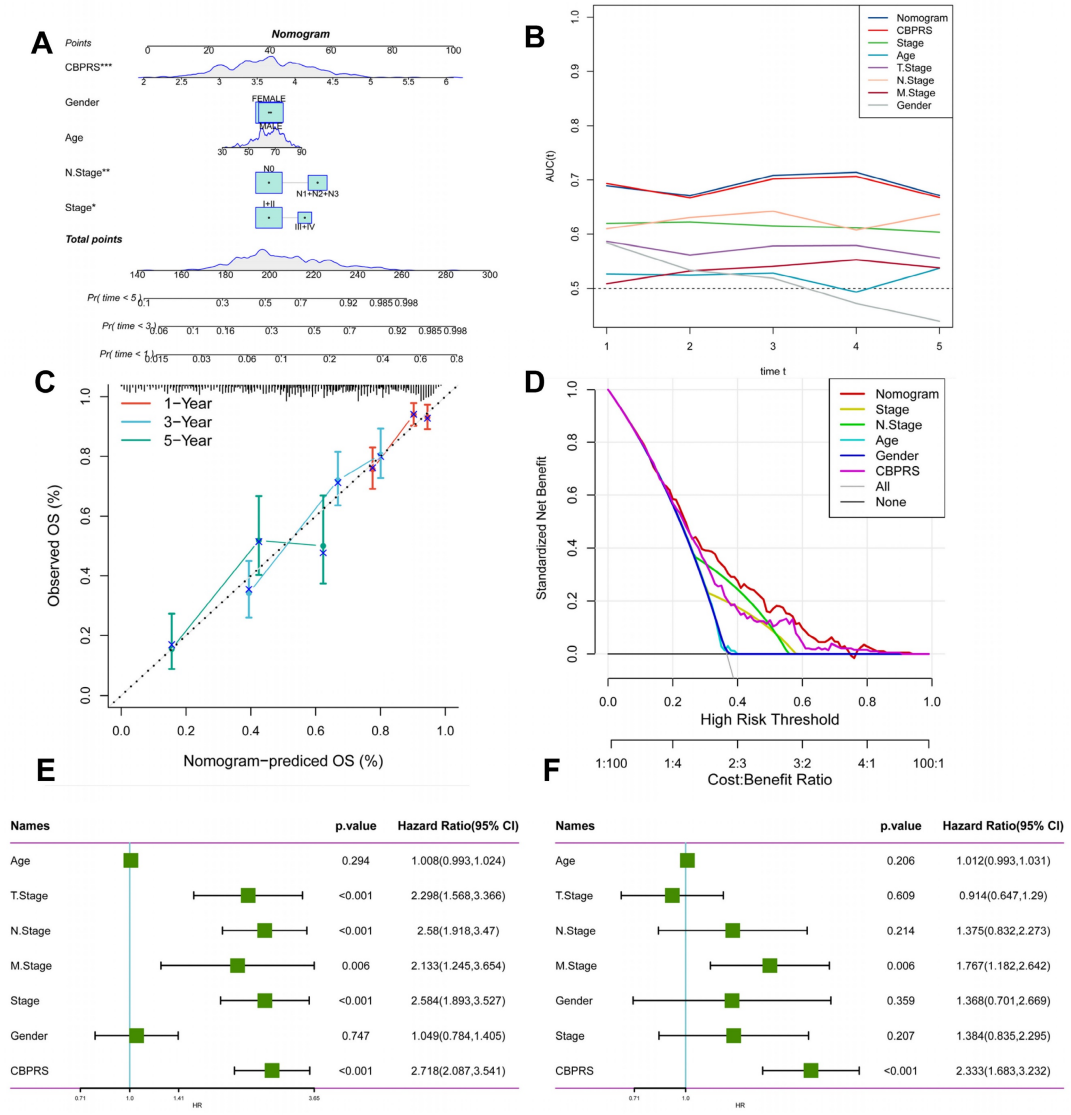
** Creation of a column chart based on the CBP model combined with clinical characteristics. (A)** nomogram plot combining Age, Gender, N-stage, Stage, and CBPRS.** (B)** Time-dependent ROC curve analysis.** (C)** Calibration curves constructed for 1-, 3-, and 5-year survival column plots.** (D)** DCA decision curve analysis.** (E)** Univariate and** (F)** multivariate COX regression analysis of characteristics and different clinical features.

We integrated risk scores and their clinical metrics to construct a nomogram as a predictor of 1-, 3-, and 5-year prognostic probability of survival (Figure 4A). TimeROC analyses in the TCGA cohort confirmed that the AUC of the column charts and riskscores exceeded that of other metrics (Figure 4B). Calibration curve analysis showed that patients' 1-, 3-, and 5-year OS prediction curves were highly similar to the ideal 45-degree calibration line, suggesting excellent stability of the column line graph (Figure 4C). Decision curve analysis (DCA) showed better predictive efficacy of nomogram and riskscore compared to other clinical characteristics (Figure 4D). In addition, to validate the reliability and clinical value of biometric traits constructed based on CBP as prognostic predictors, we compared the risk score of each LUAD patient with two common clinical indicators and observed the correlation of each factor with patient prognosis in successive univariate and post-prediction. Multivariate Cox analysis. Based on the analysis of the results, it was clear that staging, T-staging, and riskscore (P < 0.001) were all prognostic factors significantly associated with patient prognosis in the univariate cox analysis (Figure 4E). However, after multivariate cox analysis, only riskscore (P < 0.001) was significant (Figure 4F). These results suggest that our CBP model is more practical and impactful for clinical decision-making, and is more suitable as a clinical decision-making tool to predict the prognosis of patients with LUAD in clinical settings.

### Comparing the predictive effectiveness of CBPRS with existing features

**Figure 5:**
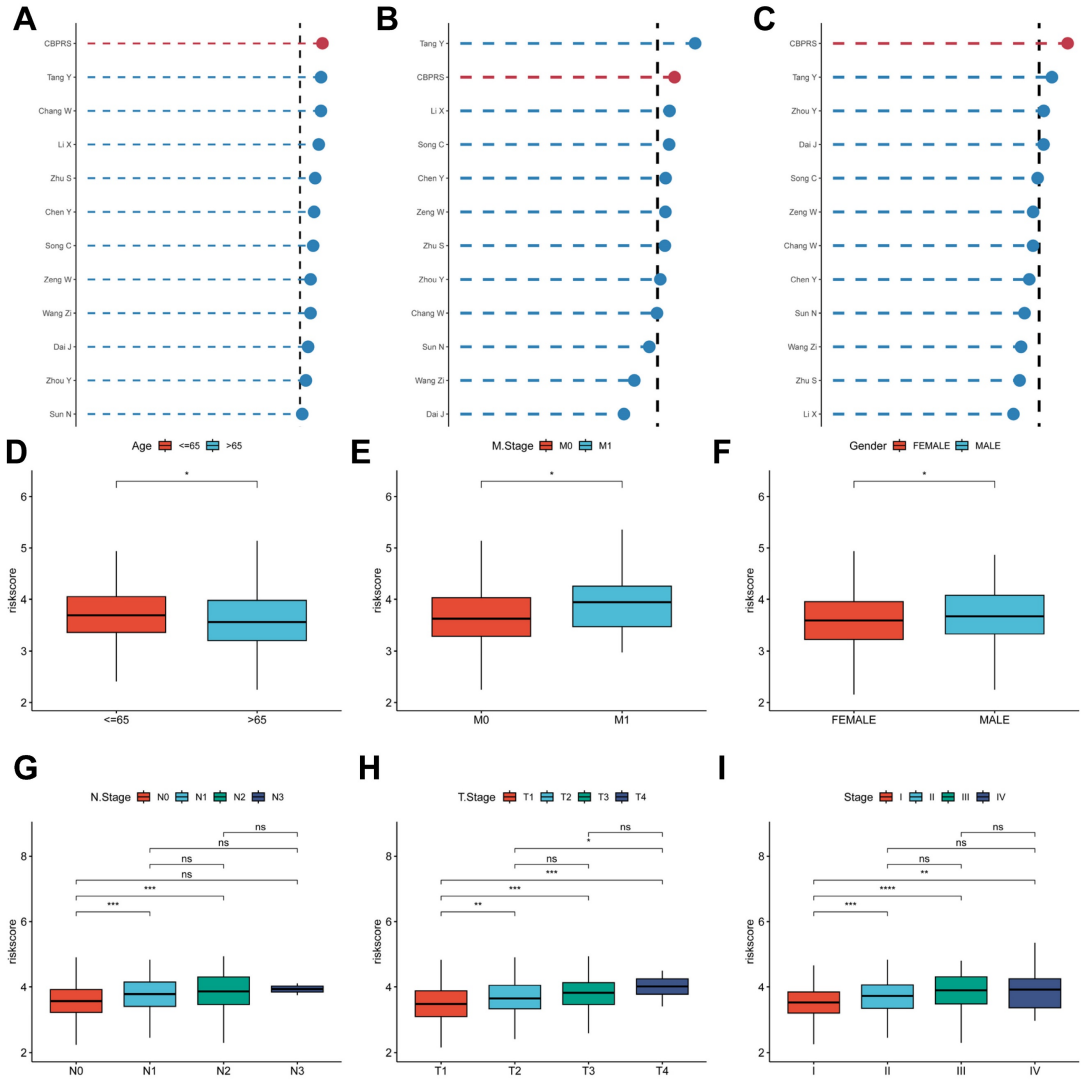
** Comparing the predictive effectiveness of CBPRS with existing features. (A-C)** Comparison between CBPRS and 10 other published models in the TCGA-LUAD, GSE31210 and GSE50081 cohorts.** (D-I)** Clinical phenotypic differences in CBPRS risk models. (D) Age (E) M.Stage (F) Gender (G) N.Stage (H) T.Stage (I) Stage

To compare the prognostic efficacy of CBPRS with existing LUAD models, we integrated 11 previous studies that used different biologically significant features, such as arginine-substituted succinate 30, copper death 31, necrotic apoptosis 32, immune activation 33, ubiquitin proteasome 34 and autophagy 35. Notably, CBPRS exhibited better C-index performance than almost all models in the TCGA-LUAD, GSE31210 and GSE50081 datasets (Figure 5A-C). In addition, Figure 5D-I, demonstrates the clinical phenotypic differences between high and low CBPRS risk models. In conclusion, these findings confirm the idea that CBPRS is a more effective prognostic model for LUAD.

### Clinical relevance and survival analysis of CBPRS in patients with LUAD

**Figure 6:**
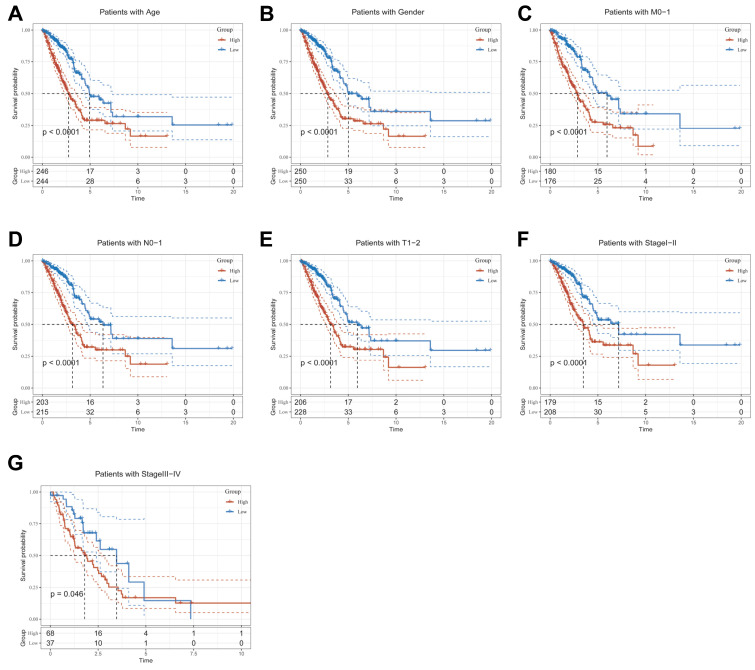
** Clinical relevance and survival analysis of CBP in LUAD patients. (A)** Age.** (B)** Gender. **(C)** Pathological M.stage.** (D)** N-stage.** (E)** T-stage (T1-2).** (F)** Stage (I-II).** (G)** Stage (III-IV).

To explore and compare the differences in individual clinical characteristics of OS between the high and low CBPRS groups, LUAD patients were categorised into five subgroups based on age, pathological stage (I-II and III-IV), gender (female and male), pathological M-stage (M0-1), N-stage (N0-N1) and T-stage (T1-2 and T3-4). Significantly, patients in the low-CBPRS group had a longer survival time compared to those in the high-CBPRS group in all subgroups (Figure 6A-G, Supplementary Figure 2A, B). These results reinforce the reliability of the CBP model as a clinical prediction tool.

### Gene set enrichment analysis

**Figure 7:**
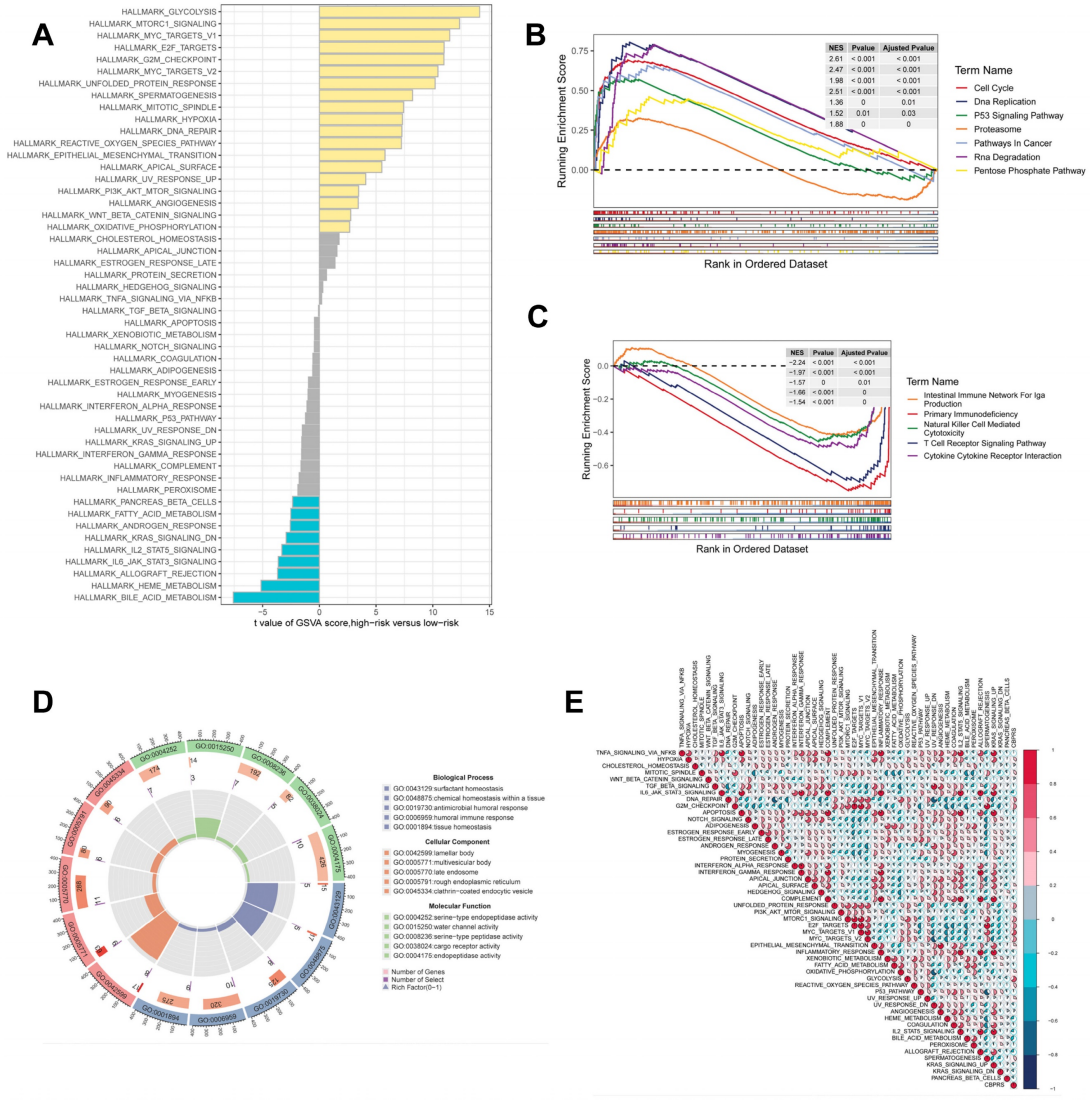
** Gene set enrichment analysis. (A)** Differences in HALLMARK pathway activity between high and low risk groups for GSVA scores.** (B)** KEGG gene set enriched in the high CBPRS group.** (C)** KEGG gene set enriched in the low CBPRS group.** (D)** Circle diagram demonstrating differential gene enrichment of the GO pathway between the two groups.** (E)** Correlation between risk scores and marker pathway activity for GSAV scores.

GSEA was used to identify KEGG gene sets enriched in both CBPRS groups. The gene set in the low CBPRS group was enriched for immune-related pathways such as the T Cell Receptor Signaling Pathway and the Intestinal Immune Network for IGA Production. In contrast, the gene set in the high CBPRS group was enriched for cell cycle- and cancer-related pathways (see Fig. 7B, C). GSVA was used to analyze the differentially enriched HALLMARK pathways between the two groups (see Figure 7A). The study found that the high-risk group was mainly associated with oncogenic pathways, while the low-risk group was mainly associated with immune-related pathways. Differential analyses were conducted on both the high-risk and low-risk groups, and the differential genes were analysed for Gene Ontology (GO) enrichment (refer to Figure 7D). Correlation analyses between CBPRS and hallmarks pathway scores supported these findings (see Figure 7E), indicating that CBPRS is closely linked to cancer-related biological processes and metabolic pathways.

### Genomic variation and intra-tumour heterogeneity in different CBPRS subgroups

**Figure 8:**
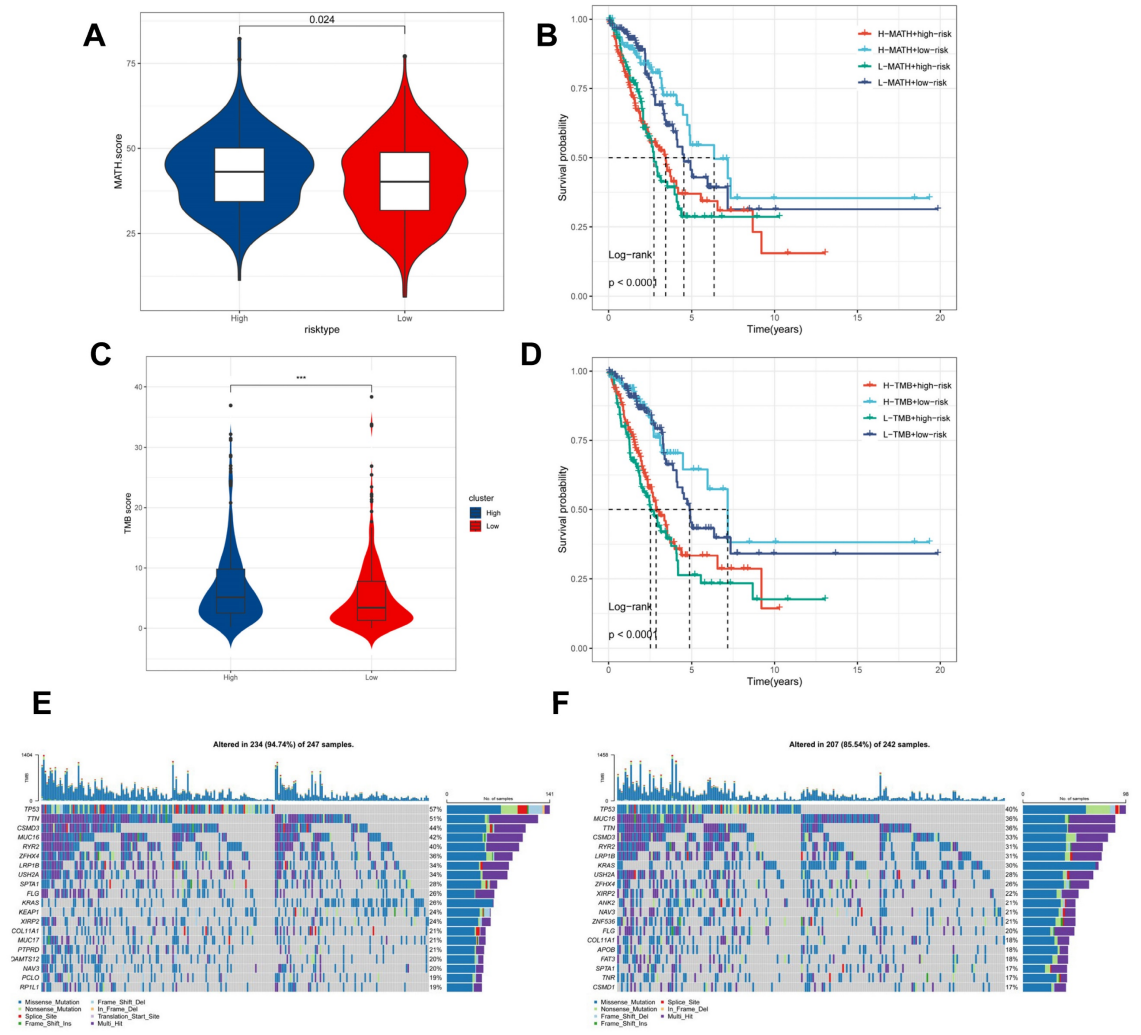
** Genomic variants and intra-tumour heterogeneity in different CBPRS subgroups. (A)** Violin plots showing the difference in mutant allele tumour heterogeneity (MATH) scores between high and low risk groups.** (B)** Kaplan-Meier curves for OS were analysed by combining the MATH score and CBPRS risk score.** (C)** Violin plot demonstrating the difference in TMB between high and low risk groups.** (D)** Kaplan-Meier curves of OS analysed by combining TMB score and CBPRS risk score.** (E)** Mutation analysis of the high-risk group.** (F)** Mutation analysis of the low-risk group.

Intra-tumor heterogeneity (ITH) is a well-known genomic feature caused by mutation 36 accumulation resulting in cancer. ITH has been shown to be associated with malignancy and increased resistance to chemotherapy 37. The mutant allele tumour heterogeneity (MATH) algorithm was used in this study to measure intratumour heterogeneity (ITH) in LUAD patients. Higher MATH scores were found to be associated with higher ITH. The high-risk group of LUAD patients had a higher MATH score (Figure 8A). The combination of ITH and CBPRS was further analysed, revealing that patients in the 'high risk + high MATH' group had a significantly worse prognosis than those in the 'low risk + low MATH' group (log-rank test, p < 0.001). This suggests that the combination of these two metrics could be a better indicator of LUAD prognosis. Figure 8B illustrates how the combination of these two metrics could better assess the prognosis of LUAD patients. Furthermore, the TMB analyses of the high and low CBPRS groups indicated significant differences. Specifically, the high CBPRS group exhibited higher TMB (Figure 8C). Additionally, the combination of TMB and CBPRS demonstrated that patients in the 'high risk + high TMB' group had a significantly worse prognosis than those in the 'low risk + low TMB' group (p < 0). The group with low TMB (log-rank test, p < 0.001) showed a better prognosis, indicating that the combination of these two metrics could be more effective in assessing the prognosis of LUAD patients (Figure 8D). It is widely accepted that genetic mutation is a prerequisite for tumourigenesis. In the TCGA database, we visualised and correlated somatic mutation data based on CBP signatures combined with high and low CBPRS groups. In the high CBPRS group, TP53 (57%), TTN (51%), and MUC16 (44%) had the highest mutation frequencies (Figure 8E, F).

### CBP risk score predicts tumour microenvironment and immune cell infiltration

**Figure 9:**
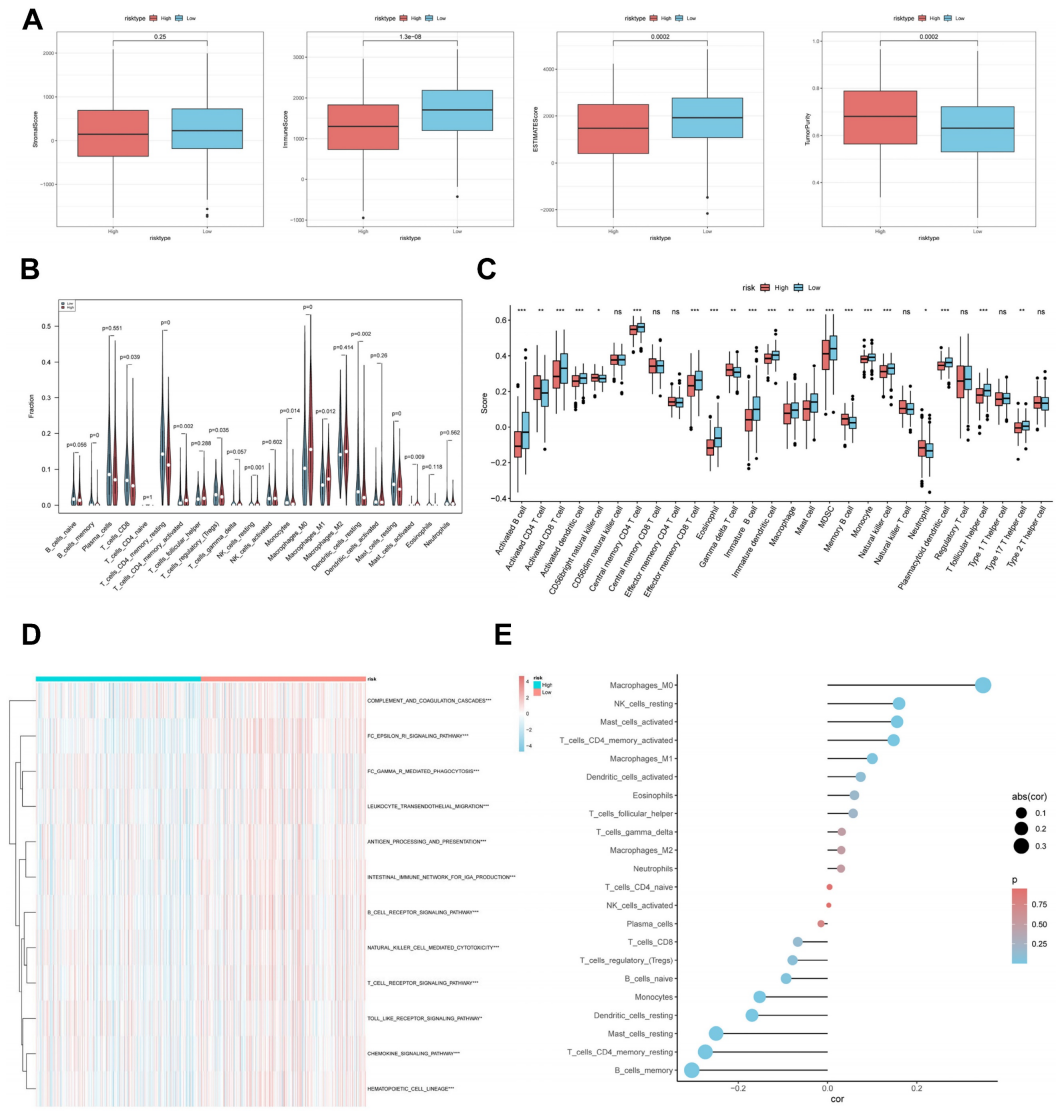
** CBP risk score predicts tumour microenvironment and immune cell infiltration. (A)** Stroma score, immunity score, ESTIMATE score and tumour purity were used to quantify different immune statuses between high and low risk groups.** (B)** Abundance of each TME-infiltrating cell type was quantified by the CIBESORT algorithm and the ssGSEA algorithm** (C)** between high and low risk groups.** (D)** The activity of immune-related pathways was significantly different between the high- and low-risk groups.** (E)** Correlation analysis of TME-infiltrating cells with CBPRS.

It has been established that interactions between cancer cells and TME are critical for tumour progression and spreading 38. To assess the immune infiltration status of the LUAD samples in this study, we used the ESTIMATE algorithm to calculate the stromal score, immune score, ESTIMATE score, and tumour purity for the CBPRS risk subgroup. The low-risk group had significantly higher immunity and ESTIMATE scores, while the high-risk group had higher tumour purity (Figure 9A). To analyse the differences in specific immune cell infiltration between the high- and low-risk groups, we quantified the abundance of immune cell infiltration in each sample using the CIBERSORT algorithm (Figure 9B). We then used the CIBERSORT results to screen for immune cell types significantly associated with CBPRS by Spearman's correlation analysis (Figure 9E). Similar results were obtained by applying the ssGSEA algorithm for validation (Figure 9C). Furthermore, using the ssGSEA algorithm, we obtained scores for immune-related pathways. It was observed that the low-risk group exhibited higher activity levels in these pathways (Figure 9D).

### Predicting and validating the efficacy of immunotherapy

**Figure 10:**
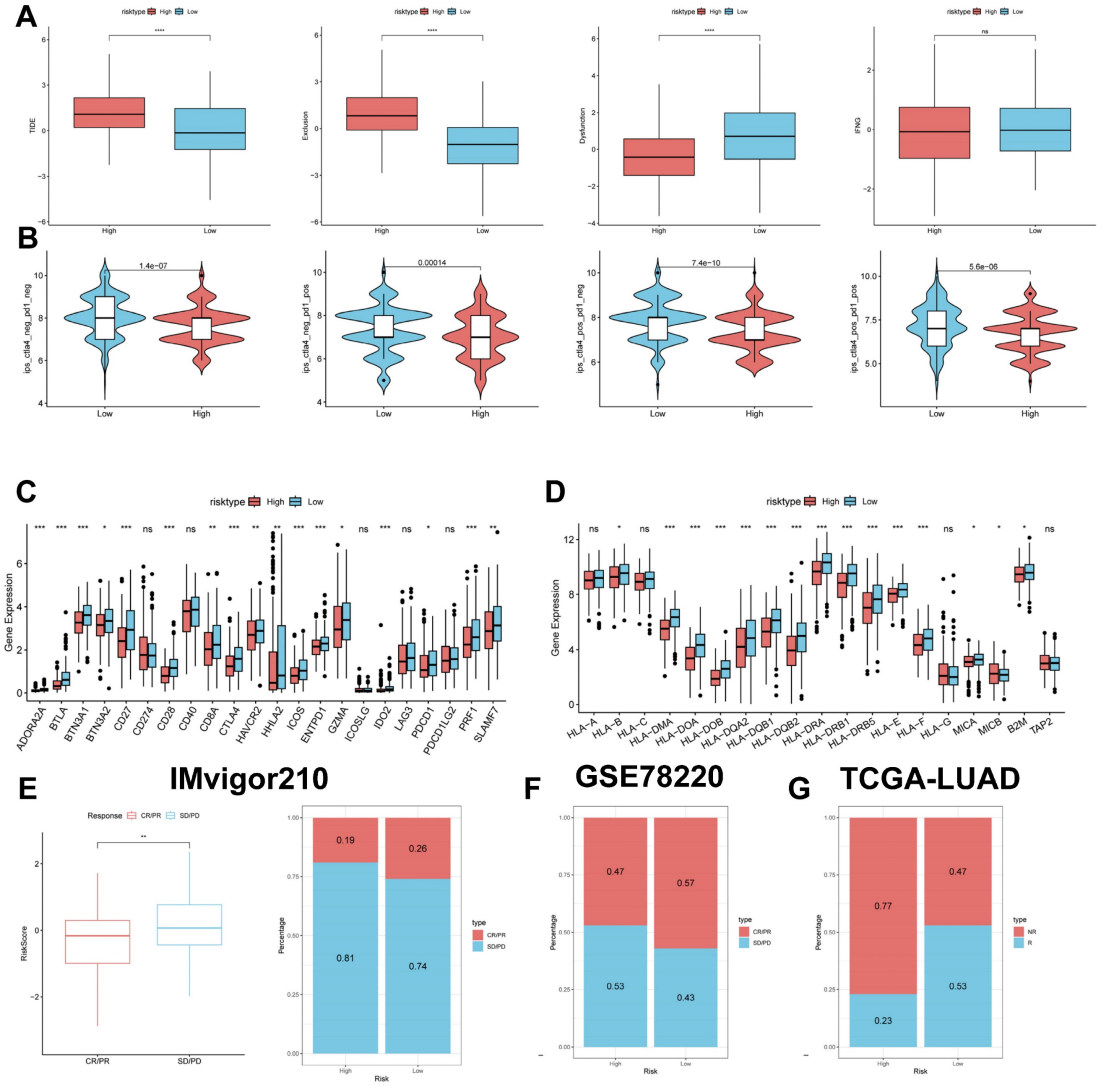
** Predicting and validating the efficacy of immunotherapy. (A)** TIDE assessment of immunotherapy escape in the high- and low-risk groups.** (B)** IPS scores in the high- and low-risk groups. **(C)** Differential expression of various immune checkpoints in the high- and low-risk groups.** (D)** Differential expression of HLA molecules in the high- and low-risk groups.** (E)** Boxplots depicting the difference in risk scores between CR/PR patients and SD/PD patients and the proportion of CR/PR or SD/PD patients receiving immunotherapy in the IMvigor210 cohort.** (F)** Proportion of CR/PR or SD/PD patients receiving immunotherapy in the high and low risk groups of the GSE78220 cohort. **(G)** Proportion of patients with R or NR who received immunotherapy in the high and low risk groups of the TCGA-LUAD cohort.

The TIDE results showed higher scores in the high-risk group, indicating that the high-risk group may have stronger immune escape (Figure 10A). To validate our results, we analysed the IPS scores obtained from the TCIA database. Higher IPS scores predicted a better response to ICI treatments, including PD-1 inhibitor and CTLA4 inhibitor treatments, in four categories: ips_ctla4_pos_pd1_pos, ips_ctla4_pos_pd1_neg, ips_ctla4_neg_pd1_pos, and ips_ctla4_neg_pd1_neg. The study results indicate that all four categories were significantly elevated in the low-risk group. This suggests that patients in the low-risk group responded better to anti-CTLA4 therapy and the combination of anti-PD-1 and anti-CTLA4 therapy than patients in the high-risk group (Figure 10B). Previous studies have reported that high expression of immune checkpoints High expression of immune checkpoints is associated with better response to immune checkpoint inhibitor (ICI) therapy 39-41.

Therefore, we analysed the differences in immune checkpoints on the basis of risk scores, and found that the expression was higher in the low-risk group (Figure 10C) 42. The molecular differences in HLA between the different groups were compared (see Figure 10D). To test the potential of risk scores in predicting immunotherapy in a real cohort, we selected two groups of patients receiving immunotherapy (IMvigor210 and GSE78220). The proportion of complete response/partial response (CR/PR) was significantly higher in the low-risk group (Figure 10C), as was the proportion of responders to immunotherapy. The number of responders to immunotherapy was also higher in the low-risk group compared to the high-risk group (Figure 10E-G). These results suggest that the low-risk group had a better response to immunotherapy.

### Correlation of CBPRS with single-cell characteristics

**Figure 11:**
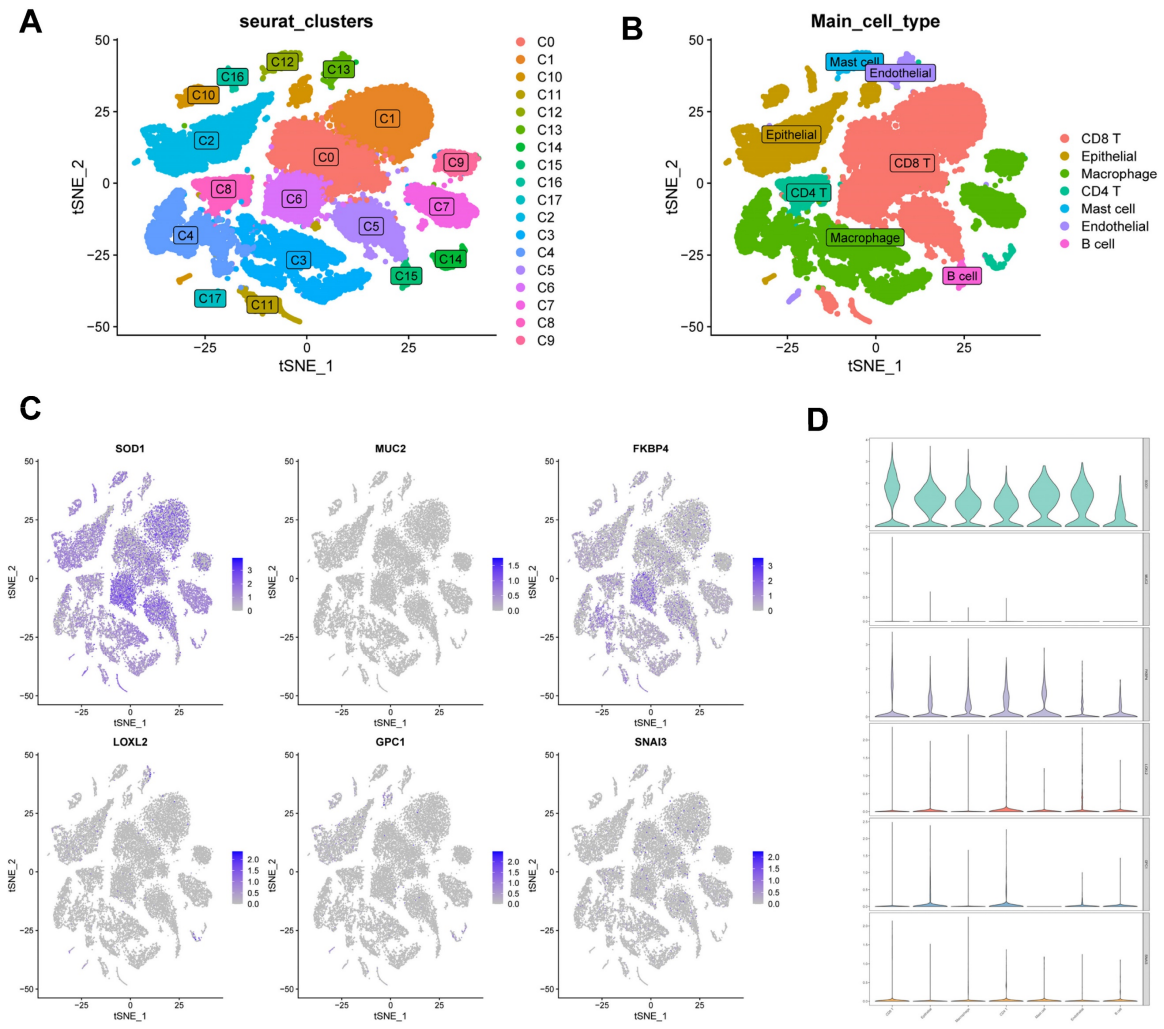
** Correlation of CBPRS with single-cell characteristics. (A)** Cells were divided into 18 independent clusters.** (B)** Cells were clustered into seven types by the tSNE dimensionality reduction algorithm, with each colour representing the phenotype of each cluster.** (C)** Feature plots showing the distri-bution of six CBP genes in various celltypes.** (D)** Violin plots showing the distri-bution of six CBP genes in various celltypes.

We used single-cell data from Philip Bischoff et al. and downscaled them using the "RunPCA" function to obtain 18 clusters (Figure 11A), which were subsequently annotated according to cell marker genes (Figure 11B). To investigate the role of CBPRS in the tumour microenvironment (TME) at the single-cell transcriptome level, we analysed the expression patterns of *SOD1*, *MUC2*, *FKBP4*, *LOXL2*, *GPC1* and *SNAI3* in different cell types (Figure 11C). The results showed that *SOD1* was expressed in most immune cells, *FKBP4* was more expressed in macrophages and CD8T cells, and the other genes were less expressed, and the violin plots mirrored these results (Figure 11D).

### Identification of key regulatory genes in the CBP model

**Figure 12:**
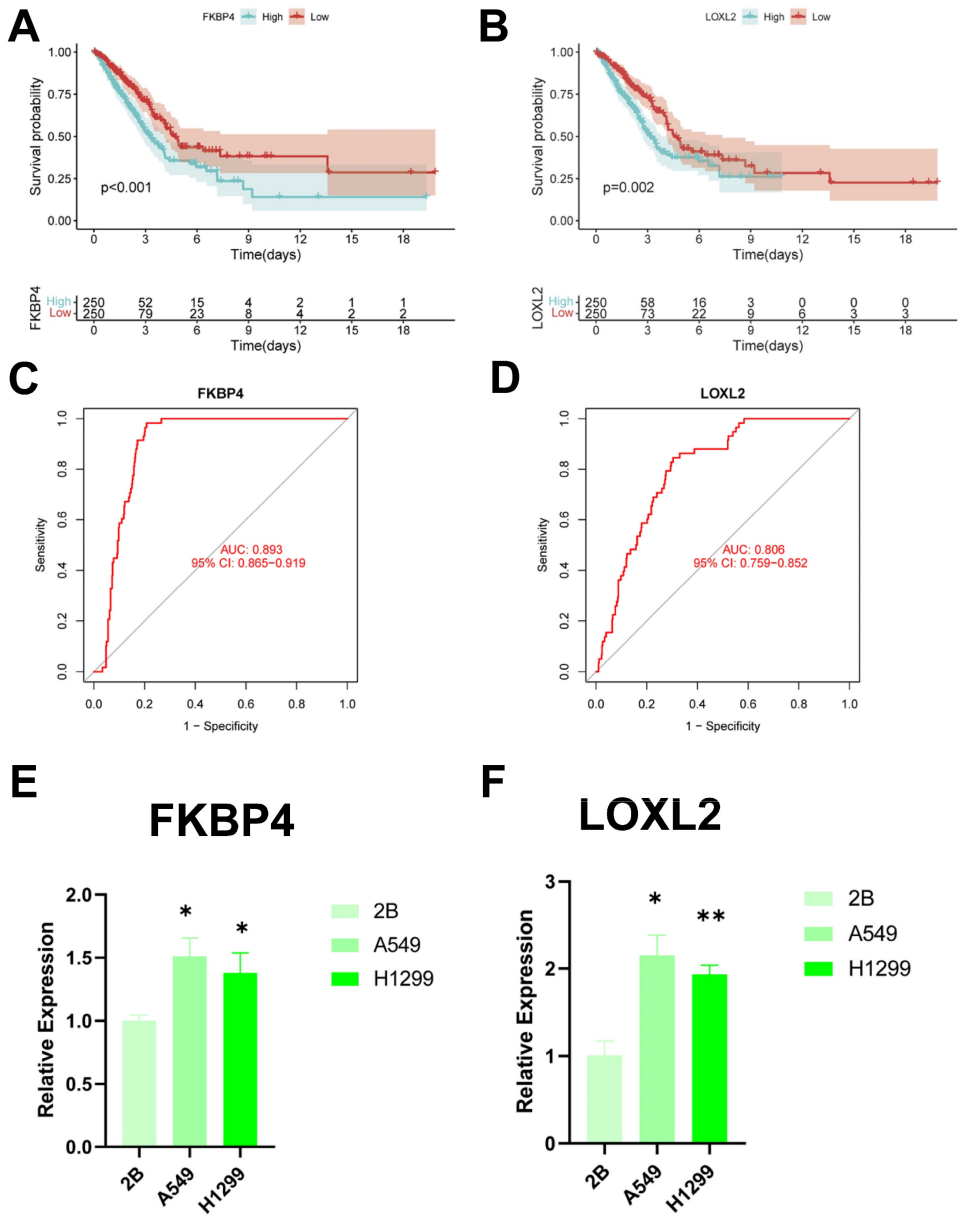
** Identification of key regulatory genes in the CBP model. (A)** KM curve of *FKBP4*.** (B)** KM curve of *LOXL2*.** (C)** ROC diagnostic curve of *FKBP4*.** (D)** ROC diagnostic curve of *LOXL2*.** (E,F)** RTq-PCR demonstrating mRNA expression levels of (E) *FKBP4*. (F) *LOXL2*.

To identify key regulators in the CBP risk subgroups, first we analysed the survival curves of these six genes and found that only *SOD1* was not significant (P > 0.05) (Figure 12A, B, **Supplementary Figure 2A-D**). In addition, we used ROC diagnostic curves to screen for key regulators, and we found that the only ones with ROC > 0.80 were *FKBP4* and *LOXL2*, and thus we considered these two genes to be key regulatory genes for CBPRS (Figure 12C, D, Supplementary **Figure 3A-D**). Finally, we assessed the expression of the two core genes in CBPRS in three cell lines, including one normal cell line (2B) and two lung adenocarcinoma cell lines (A549 and H1299) (Figure 12E, F). The results showed that FKBP4 and LOXL2 expression was significantly upregulated in the tumour cell lines.

## Discussion

Despite significant efforts to develop comprehensive treatment strategies, the prognosis for patients with LUAD remains poor, with a 5-year survival rate of 15 per cent 5. Exploring potential mechanisms and prognostic biomarkers may help precision medicine for cancer patients. Further discovery of potential mechanisms of tumour progression could lead to the development of new therapeutic strategies for lung adenocarcinoma.

Cu, as a trace element, is involved in a wide range of biological activities and plays a vital role in living matter. Cu is also associated with a variety of cellular processes, including mitochondrial respiration, antioxidant defence, redox signalling, kinase signalling, autophagy and protein quality control 43. However, disruption of copper homeostasis can lead to accumulation of reactive oxygen species and proteasome inhibition, which can cause cytotoxicity 44. Abnormally elevated systemic copper levels within tumours of cancer patients promote tumourigenesis, angiogenesis, tumour metastasis and recurrence of many cancers 45. Recent studies have identified copper death as a key regulator of cancer progression 46 and that the profile of copper death is strongly associated with the prognosis of patients with a variety of cancers 47. The characteristics of copper death are closely related to the prognosis of patients with a variety of cancers. As important transport proteins and downstream effectors of copper, copper-binding proteins have also been reported to be key regulators of various tumours and are strongly associated with the prognosis of cancer patients 48. However, little is known about the role of copper-binding proteins in LUAD.

This study presents a bioinformatics analysis of copper-binding proteins in LUAD using data from the TCGA and GEO cohorts. The analysis reveals a profile of copper-binding protein-related genes and TME features in LUAD, demonstrating the genetic and transcriptional variation of CBPRGs in LUAD.

Additionally, a new prognostic model was created by screening six modelled genes using Lasso regression analysis and one-way COX risk regression analysis. Significant prognostic differences were found between the two groups, demonstrating the independent predictive value of the CBP traits created for LUAD. ROC curve and calibration curve analyses showed the superior predictive efficacy of the CBP traits for patient prognosis. Additionally, the column-line plots demonstrate the superiority of the CBP model compared to other clinically used indications. We compared our CBPRS model with 11 previously published models. The results demonstrate its good predictive efficacy.

Then, to gain more insight into the immunological properties of CBPRS, we examined mutations in different CBPRS populations. As previously reported, missense variants were the most prevalent, followed by nonsense variants and shift deletions 49. *TP53* mutations were more common in the high CBPRS group than in the low CBPRS group (57% vs. 40%), with the largest difference in mutation frequency between the groups. *TP53* mutations are not only commonly inherited in cancer, but also lead to aggressive malignancies and a poorer prognosis for patients 50, 51. Through the p53/TGF-b signalling pathway, *TP53* can influence the cancer cell cycle. Finally, a better understanding of TME may help in the development of new therapies for LUAD or in repairing TME to improve the effectiveness of immunotherapy. The composition of some immune cells differs between the two CBPRS groups; M0 and M1 macrophages are more common in the high CBPRS group, while cytotoxic CD8 T cells are more abundant in the low CBPRS group. Numerous studies have shown that a dense infiltration of T cells, especially cytotoxic CD8 T cells, is a marker of good prognosis 52-54. In addition, based on pathway enrichment, we found that the low CBPRS group had stronger immune pathways, whereas the high CBPRS group contained more immunosuppressive cells and oncogenic signals, as well as tumour and metastasis-associated signals, suggesting that the high CBPRS group exhibited immunosuppression and active tumour progression.

IPS data downloaded from TCIA can provide a predictive score for assessing a patient's response to immunotherapy 55, 56. The study suggests that patients with low CBPRS may have a more favourable response to ICI therapy, as indicated by the higher IPS in the low CBPRS group. Additionally, the study found that CBPRS, which has not been previously detected in LUAD, may strongly correlate with immune infiltration in LUAD, indicating the potential relevance of CBPRS in assessing response to immunotherapy. For patients diagnosed with early-stage LUAD, surgical treatment, ablation, or liver transplantation are effective therapeutic options that can significantly improve patient survival time. In contrast, for patients with advanced LUAD, systemic therapy is the only viable option to improve survival. In addition to immunotherapy-related drugs, we also use certain chemotherapeutic drugs. Generally, the low-CBPRS group responds better to treatment than the high-CBPRS group, resulting in improved survival time for patients with LUAD. This is supported by the TIDE results.

Based on these findings, we conclude that CBPRS is a good model for predicting survival time in LUAD patients and is closely related to the immune microenvironment. An in-depth study of CBPRS will be beneficial for treating patients with lung adenocarcinoma, thus improving the efficacy of immunotherapy. Next, six genes comprise CBPRS: *FKBP4*, *SOD1*, *MUC2*, *LOXL2*, *GPC1*, and *SNAI3*. We screened the key CBPRS regulatory genes *FKBP4* (FKBP Prolyl Isomerase 4) and *LOXL2* (Lysyl Oxidase Like 2) by ROC curve. *FKBP4* has a potential role in tumourigenesis and is considered a possible biomarker. *FKBP4* is expressed in most tissues, with the lowest expression in breast, bladder and testis 57, 58. *FKBP4* expression is elevated in several cell lines of hormone-dependent cancers, including breast cancer cell lines 59, 60 and prostate cancer cell lines 61 *FKBP4* expression is elevated in several hormone-dependent cancer cell lines, including breast and prostate cancer cell lines. Moreover, *FKBP4* expression was higher in breast cancer tissue and pre-invasive breast cancer than in normal breast tissue 60, 62. In prostate biopsy tissues 63 and liver cancer tissues 64 similar observations were made, suggesting that *FKBP4* may be a potential biomarker for tumours. Up-regulation of *LOXL2* leads to metastasis in the tumour microenvironment, which results in invasive migration 65. Although some genes have been studied for their regulatory role in cancer, few researchers have systematically evaluated their prognostic value in LUAD. Copper-binding proteins have been less studied in lung adenocarcinoma, so we hope that the establishment of CBPRS will be used to improve the clinical management of lung adenocarcinoma patients.

Although the CBP model we constructed is excellent in identifying the immune status of patients and predicting their prognosis, it is important to acknowledge some limitations in our follow-up study and address them appropriately. Firstly, the TCGA-LUAD dataset we included was based on public database data, which may introduce bias between the predictions and the actual situation. Although efforts have been made to prevent it, more data from LUAD patients is required to validate the model's utility and the accuracy of immunotherapy predictions.

## Conclusion

As demonstrated for the first time, the CBP model is a novel predictive biomarker and a possible therapeutic target for LUAD patients. It has better predictive efficacy compared to other published articles. Additionally, the CBP model can characterise the immune environment of LUAD patients and estimate the prognosis of LUAD patients, providing physicians with a new approach to treating lung adenocarcinoma patients.

## Supplementary Material

Supplementary figures.

Supplementary tables.

## Figures and Tables

**Table 1:** TCGA-LUAD Clinical characteristics.

Characteristics	High (N=250)	Low (N=250)	P-value
Age			
<65	134 (53.6%)	103 (41.2%)	0.009
>=65	112 (44.8%)	141 (56.4%)	
Unknown	4 (1.6%)	6 (2.4%)	
Gender			
Male	128 (51.2%)	102 (40.8%)	0.025
Female	122 (48.8%)	148 (59.2%)	
Stage			
I	108 (43.2%)	160 (64.0%)	2.7e-05
Ii	71 (28.4%)	48 (19.2%)	
Iii	51 (20.4%)	29 (11.6%)	
Iv	17 (6.8%)	8 (3.2%)	
Unknown	3 (1.2%)	5 (2.0%)	
T stage			
T1	70 (28.0%)	97 (38.8%)	0.003
T2	136 (54.4%)	131 (52.4%)	
T3	29 (11.6%)	16 (6.4%)	
T4	14 (5.6%)	4 (1.6%)	
Tx	1 (0.4%)	2 (0.8%)	
N stage			
N0	144 (57.6%)	180 (72.0%)	0.001
N1	59 (23.6%)	35 (14.0%)	
N2	43 (17.2%)	26 (10.4%)	
N3	2 (0.8%)	0 (0%)	
Nx	8 (2.0%)	2 (2.0%)	
Unknown	2 (0.8%)	9 (3.6%)	
M stage			
M0	163 (65.2%)	169 (67.6%)	0.065
M1	17 (6.8%)	7 (2.8%)	
Unknown	70 (28.0%)	74 (29.6%)	
