# Supplementary material for: Copper-binding protein modelling by single-cell transcriptome and Bulk transcriptome to predict overall survival in lung adenocarcinoma patients
Source: J Cancer. 2024 Mar 17;15(9):2659–77. doi: 10.7150/jca.94588 (PMC10988321; doi:10.7150/jca.94588)
Supplement: Supplementary file 1 — Supplementary figures. [file jcav15p2659s1.pdf]

# Supplementary Figures:

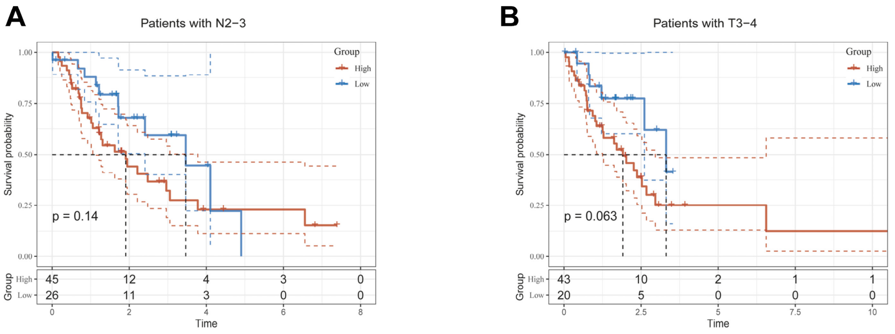


**Supplementary Figure.1**

**Clinical features of CBPRS**

(A)km curves for patients N2 and N3. (B) km curves for patients T3 and T4.

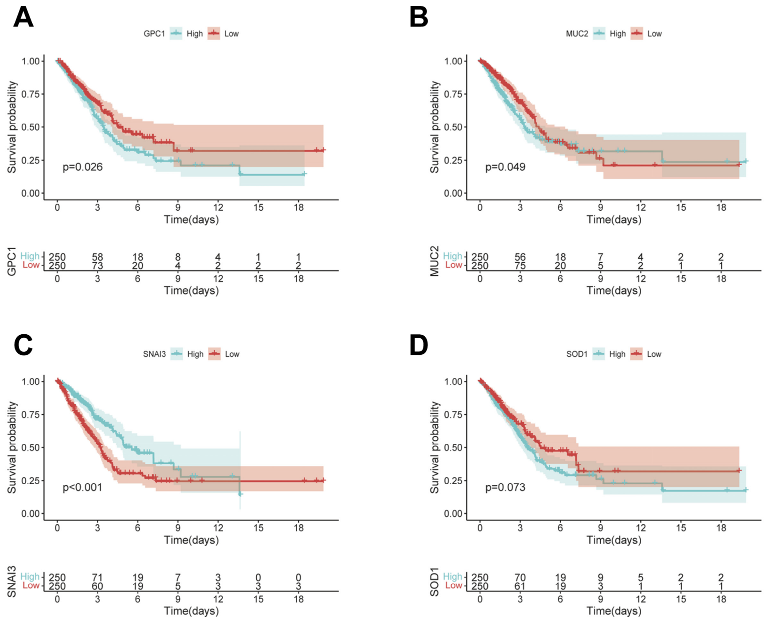


**Supplementary Figure.2**

**KM survival curves for the CBPRS genes**

KM survival curves for the CBPRS genes including GPC1(A), MUC2(B), SNAI3(C), SOD1(D).

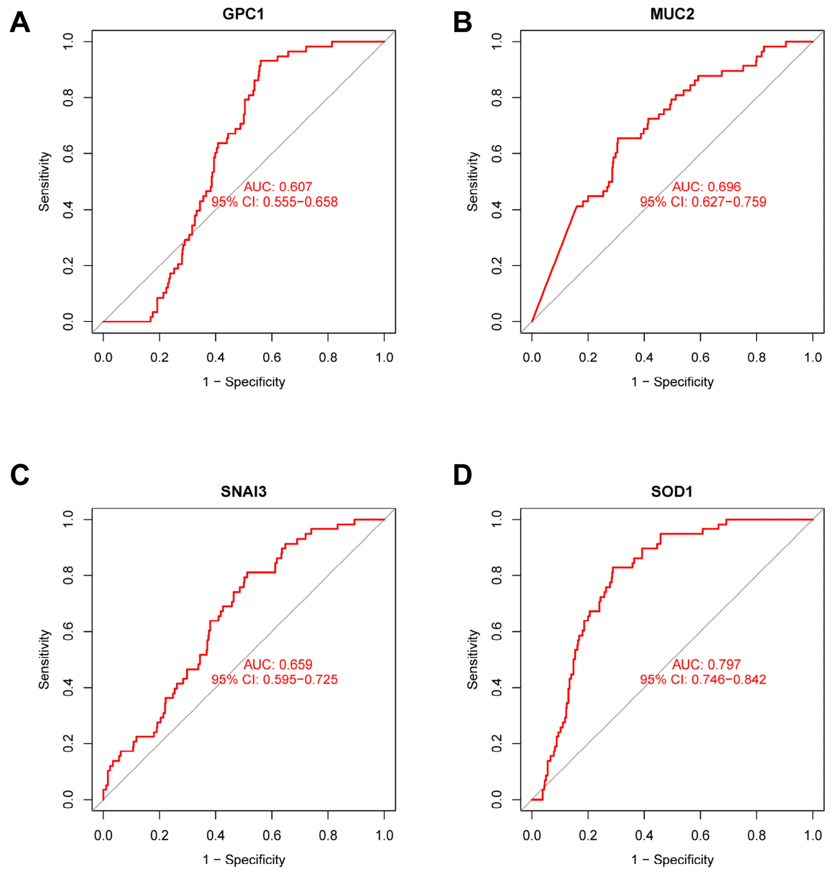


**Supplementary Figure.3**

**ROC diagnostic curve**

ROC diagnostic curve for the CBPRS genes including GPC1(A), MUC2(B), SNAI3(C), SOD1(D).
